# Comment: In Response to “CD9 Is a Very Helpful Marker for Discriminating AML-M3 from HLA-DR-Negative Non-M3 AML”

**DOI:** 10.4274/tjh.galenos.2020.2020.0572

**Published:** 2020-11-19

**Authors:** Smeeta Gajendra

**Affiliations:** 1 Laboratory Oncology Unit, Dr. B.R.A. IRCH, AIIMS, New Delhi, India

**Keywords:** Acute promyelocytic leukemia, Flow cytometry, CD9, CD18

## To the Editor,

I read the letter “CD9 Is a Very Helpful Marker for Discriminating AML-M3 from HLA-DR-Negative Non-M3 AML” by Satlsar et al. [1], published in this journal. The manuscript is well written with description of a very informative topic of expression pattern of CD9 in acute promyelocytic leukemia (APL). There is bright homogeneous expression of CD9 in APL, whereas the expression is dimmer and heterogeneous in non-APL  cases. CD9 is a tetraspanin molecule that is expressed on a wide variety of hematopoietic cells as precursor B cell, megakaryocytes, and certain acute myeloid leukemias. CD9 is associated with poor prognosis in ALL and good prognosis in AML cases. CD9 can be used as a biomarker  and can be considered as therapeutic target. APL has distinct morphologic, biologic, and clinical features. The diagnosis is predominantly based on morphology which is characterized by presence of abnormal promyelocytes with bilobed nuclei and frequent Auer rods and a cytogenetic/molecular hallmark of t(15;17)(q22;q21) *PML-RARA.* Multiparameter flow cytometry evolved as a rapid diagnostic tool along with morphology for early detection and immediate starting of therapy to avoid life threatening complications. On flow cytometry, these abnormal promyelocytes have a teardrop pattern with high SCC in CD45-SSC plots due to prominent granulations which mimic the position of granulocytes but lack CD15, CD16, and CD11c, which would be present on neutrophils. These are predominantly CD34-HLA DR negative with myeloperoxidase, CD13, CD33, and CD117 positive. Aberrant expression of CD2 and CD56 is commonly seen. They characteristically lack expression of the β2-integrins CD11a, CD11b, CD11c, and CD18 in 100% cases [2,3]. However, CD11a and CD18 are absent in 39% and 45%  of non-APL cases, respectively [4]. Many studies have shown that the absence of CD34 and HLA-DR is not specific for APL and about 50% of HLA-DR negative cases include other AML subtypes [5]. HLA-DR and CD34 negativity is a distinctive feature of “AML-cuplike” with *FLT3*-ITD [6] and in AML-M1 and AML-M2 subtypes with *NPM1* mutation [7]. CD9 is expressed in around 40% cases of AML [8]. Liu et al. [9] showed a significant expression of CD9 in AML with *NPM1* mutation. Thus, as a single marker presence of CD9 or absence of HLA-DR, CD18, or CD11a loses specificity in diagnosis of APL. Therefore, a combination of cMPO, CD34, HLADR, CD117, CD33, CD15, CD11c, CD64, CD9, and CD18 can diagnose APL with high specificity and sensitivity by excluding other hypergranular AML as few cases of *NPM1-*positive AML mimicking APL on morphology and also in cases showing CD34 and HLA-DR negativity on flow cytometry, that can pose a diagnostic dilemma. However, definitive diagnosis of APL requires cytogenetic or molecular study.
