# Supplementary material for: Comment: In Response to “CD9 Is a Very Helpful Marker for Discriminating AML-M3 from HLA-DR-Negative Non-M3 AML”
Source: Turk J Haematol. 2020 Nov 19;37(4):289–90. doi: 10.4274/tjh.galenos.2020.2020.0572 (PMC7702650; doi:10.4274/tjh.galenos.2020.2020.0572)
Supplement: Related texts

# Reply: Authors' Response

In reply to: Comment: In response to “CD9 Is a Very Helpful Marker for Discriminating AML-M3 from HLA-DR Negative Non-M3-AML”

## To the Editor,

We are proud that our research has received attention. This research is a part of continuous research for precise flow cytometry detection of acute promyelocytic leukemia [1]. The use of myeloid markers such as CD13, CD33, CD64, CD117, CD34, HLA-DR, and CD11b is essential for diagnosis of APL, as we mentioned in the last paragraph. In clinical practice differential diagnosis of HLA-DR-negative AML from APL is very crucial and using different markers can be very helpful in these cases. Our aim is to introduce CD9 further to increase the specificity and sensitivity of flow cytometry, which can be used with other markers as complementary  marker.

Best Regards,

Esmaeil Shahabi Satlsar, Mohammad Mosleh, Mahdieh Mehrpouri

## References

[1] Mosleh M, Mehrpouri M, Ghaffari S, Saei Z, Agaeipoor M, Jadali F, Satlsar ES, Gholampour R (2020). Report of new six-panel flow cytometry for early differential diagnosis of APL from HLA-DR negative Non-APL leukemia. Scand J Clin Lab Invest.
